# Nonparametric Expectile Shortfall Regression for Complex Functional Structure

**DOI:** 10.3390/e26090798

**Published:** 2024-09-18

**Authors:** Mohammed B. Alamari, Fatimah A. Almulhim, Zoulikha Kaid, Ali Laksaci

**Affiliations:** 1 Department of Mathematics, College of Science, King Khalid University, Abha 62529, Saudi Arabia; malamari@kku.edu.sa (M.B.A.); zqayd@kku.edu.sa (Z.K.); alikfa@kku.edu.sa (A.L.); 2 Department of Mathematical Sciences, College of Sciences, Princess Nourah Bint Abdulrahman University, P.O. Box 84428, Riyadh 11671, Saudi Arabia

**Keywords:** financial risk, complete consistency, expected shortfall, functional data, kernel method, expectile regression, quantile regresion

## Abstract

This paper treats the problem of risk management through a new conditional expected shortfall function. The new risk metric is defined by the expectile as the shortfall threshold. A nonparametric estimator based on the Nadaraya–Watson approach is constructed. The asymptotic property of the constructed estimator is established using a functional time-series structure. We adopt some concentration inequalities to fit this complex structure and to precisely determine the convergence rate of the estimator. The easy implantation of the new risk metric is shown through real and simulated data. Specifically, we show the feasibility of the new model as a risk tool by examining its sensitivity to the fluctuation in financial time-series data. Finally, a comparative study between the new shortfall and the standard one is conducted using real data.

## 1. Introduction

With the huge development and the progress of computer and data science, the statistical modeling of complex and unstructured data is becoming indispensable. In this context, the functional statistics constitutes a good mathematical tool to fit this situation. At this stage, we aim in this contribution to develop a new metric of risk management. More precisely, instead of the standard expected shortfall, we define a new expected shortfall regression (ESR) using the conditional expectile. In fact, the use of the expectile instead of the quantile in the ESR is motivated by the principal feature of the expectile that is very sensitive to the outliers or the extreme risk.

From a historical point of view, the expectile function was introduced by [1]. It constitutes a good alternative to the quantile. In financial time-series analysis, the use of the expectile instead of the VaR function is motivated by its high sensitivity to the outliers, which increase its ability to fit the financial risk. At this stage, the expectile function has gained popularity in risk analysis management. For more motivations on this model in financial risk management, we refer the reader to [2,3,4,5]. Furthermore, the expectile function has been used for other statistical modeling, including the outliers testing (see [6]) or the heteroscedasticity detection (see, for instance, [7,8]). Concerning the use of the expectile in the regression analysis, we cite [9] for the multivariate case and [10] for the functional case. The authors of this last cited work have obtained the asymptotic properties of the nonparametric estimation of the expectile regression with a functional covariate. Alternatively, the functional version of the parametric expectile regression was studied by [11]. They used the reproducing kernel Hilbert space structure to construct their estimator. They obtained the asymptotic upper and lower bounds of the convergence rate. In parallel, the shortfall function was introduced by [12]. The use of this risk metric in financial time-series data is motivated by its coherency property. We return to [12] for a comparative study between the Value at Risk (VaR) and expected shortfall model (ES) in financial time-series analysis. The authors of this cited paper have proved that the VaR is unusable when the profit–loss is not Gaussian. From an analytical point of view, the estimation of the ES model can be performed by multiple ways including parametric, semi, or nonparametric algorithms. The parametric approaches were used by [13,14,15]. Meanwhile, the first results in the nonparametric techniques were obtained by [16]. He used the kernel method to construct an estimator of the ES-model. Ref. [17] established the asymptotic distribution of the kernel estimator of the ES model. Using the Bahadur representation, the authors of [18] have constructed an alternative estimator of the ES-model. Ref. [19] has studied the functional version of the Nadaraya–Watson estimator of the conditional ES model (CESM) under the mixing assumption. They proved that the constructed estimator almost completely converges. Alternative functional time-series data were developed by [20]. In particular, they obtained the almost complete consistency of the kernel estimator of the CESM under the quasi-associated dependency. While in all the previous cited work, the expected loss of the shortfall is defined by the VaR level, in this paper, we introduce an alternative risk threshold that is the expectile regression.

As mentioned below, the main aim of the present contribution is to develop a new risk metric based on the expectile regression. Specifically, we define the expected shortfall with respect to the tail expectile. Such a new risk metric accumulates the advantages for two functions. Indeed, it is well known that the expectile is an elicitable and coherent risk metric. Moreover, it is very sensitive to the magnitude of the lower tail, unlike the VaR, which is not influenced by the outliers. In parallel, the ES model fulfills the condition of spectral risk measures (see [21]). Thus, the ES model based on a tail expectile improves significantly the risk management. The particularity of the present contribution is the treatment of this model using the functional time-series structure. Thus, the principal achievement of this paper is the construction of a computational kernel estimator and the study of its asymptotic property using the mixing assumption. It should be noted that the functional time-series case is more realistic than the independent functional data. The practical implementation of this risk metric has been evaluated using artificial and real data. To the best of our knowledge, no attempt has been made so far to estimate the functional EES regression based on the tail expectile. We may refer to [22,23,24,25,26,27] for more recent advances in ftsa.

The paper is organized as follows. We present our risk metric as well as its estimator in Section 2. Section 3 is dedicated to introducing the functional time-series framework. The almost complete convergence of the constructed estimator is shown in Section 4. Section 5 is devoted to discussing some of the computation ability of the estimator over artificial and areal data applications. Finally, the proofs of the auxiliary results are given in the Section 6.

## 2. Model and Estimator

Let $\left(X_{1},Y_{1}\right),\ldots \left(X_{n},Y_{n}\right)$ be *n* pairs of random pairs in F×IR which are identically distributed as (X,Y). Moreover, we suppose that the regular version of the conditional distribution of *Y* given *X* exists. The standard ES regression is defined through the tail-quantile as

$$\mathrm{for}\;z\in F,\;\mathrm{by}\;RES_{p}\left(z\right)=I\;E\left[Y|Y>RVaR_{p}\left(z\right),X=z\right],\;p\in \left(0,1\right)$$

where $RVaR_{p}$ is the quantile regression of order 1−p. So, alternatively to this tail quantile, we introduce the ES regression using the tail expectation, which is defined as

$$\mathrm{for}\;z\in F,\;\mathrm{by}\;REX_{p}\left(z\right)=I\;E\left[Y|Y>REXP_{p}\left(z\right),X=z\right],\;p\in \left(0,1\right)$$

where ${REXP}_{p}$ is the expectile regression. The latter is defined by

$${REXP}_{p}\left(z\right)=\arg\min_{t\in I\;R}\left\{I\;E\left[p{\left(Y-t\right)}^{2}{𝟙}_{\left\{(Y-t)>0\right\}}|X=z\right]\right.$$

$$+\left.I\;E\left[\left(1-p\right){\left(Y-t\right)}^{2}{𝟙}_{\left\{(Y-t)\leq 0\right\}}|X=z\right]\right\}\;p\in \left(0,1\right).$$

where $1_{A}$ is the indicator function of the set *A*.

It worth noting that the replacement of $RVaR_{p}$ by ${REXP}_{p}$ permits remedying the lack of risk insensitivity of $RVaR_{p}$ to the extreme values. This characteristic is very important in practice because the catastrophic losses are located at the extreme values.

Now, for the estimation step, we assume that **F**(·) is a known measurable function and $r=r_{n}$ is a positive sequence of real numbers tending to zero as *n* tends to infinity. Next, the estimation procedure involves two steps. In the first step, we start by estimating the expectile regression ${REXP}_{p}$. The latter is estimated by ${\hat{REXP}}_{p}$, as a kernel estimator of ${\hat{REXP}}_{p}$ defined as the root of

$$\;\hat{G}\left({\hat{REXP}}_{p}\left(z\right);x\right)=\frac{p}{1-p}\;p\in \left(0,1\right)$$

with

$$\tilde{G}\left(t;x\right)=\frac{-\sum_{i=1}^{n}F_{ni}\left(z\right)\left(Y_{i}-t\right){𝟙}_{\left\{\left(Y_{i}-t\right)\leq 0\right\}}}{\sum_{i=1}^{n}F_{ni}\left(z\right)\left(Y_{i}-t\right){𝟙}_{\left\{\left(Y_{i}-t\right)>0\right\}}},\;\;\;\mathrm{for}\;\;t\in I\;R,$$

where

$$F_{ni}\left(z\right)=\frac{F_{i}}{\sum_{i=1}^{n}F_{i}}\;\mathrm{and}\;F_{i}=F\left[r^{-1}d\left(z,X_{i}\right)\right].$$

The second step is the estimation of the ES regression. Naturally, the ES regression is estimated by

(1) $$\hat{REX_{p}}\left(z\right)=\frac{\sum_{i=1}^{n}F\left[r^{-1}d\left(z,X_{i}\right)\right]Y_{i}1_{Y_{i}>{\hat{REXP}}_{p}\left(z\right)}}{\sum_{i=1}^{n}F\left(r^{-1}d\left(z,X_{i}\right)\right)},\;p\in \left(0,1\right)$$

The main purpose of the theoretical section of this work is to establish the almost complete consistency of the estimator $\hat{REX_{p}}\left(\cdots \right)$ to $REX_{p}\left(\cdot \right)$ using strong functional time-series data. For the reader not familiar with this aspect of functional time-series data analysis, we devote the rest of this section to recalling the definition of the strong mixing assumption property, which requires the introduction of the following notations. Firstly, we consider $\left\{Z_{i},i=1,2,\ldots \right\}$ to be a strictly stationary sequence of random variables, and we denote by $S_{i}^{k}\left(Z\right)$ the σ−algebra generated by $\left\{Z_{j},i\leq j\leq k\right\}.$ Secondly, for a positive integer *n*, we define

$$\begin{array}{ccc} \alpha \left(n\right)=\sup\{ & | & I\;P\left(A\cap B\right)-I\;P\left(A\right)I\;P\left(B\right)|:A\in S_{1}^{k}\left(Z\right)\mathrm{and}B\in F_{k+n}^{\infty }\left(Z\right),\;\\ & & \left.k\;\mathrm{is}\;\mathrm{strictly}\;\mathrm{positive}\;\mathrm{integer}\;\;\right\}. \end{array}$$

So, the sequence $\left\{Z_{i},i=1,2,\ldots \right\}$ is said to be α-*mixing (strong mixing) if the mixing coefficient* α(n)→0 *as* n→∞. It is well documented that this condition is verified by many processes including the usual ARMA processes (with innovations satisfying some existing moment conditions) (see [28]), the EXPAR models (see [29]), the ARCH models (see [30]), and the GARCH model (see [31]), among others.

## 3. Main Asymptotic Result

Before stating the asymptotic properties of the estimator $\hat{REX_{p}}$, we need to introduce some notations and assumptions. Firstly, we set by $C_{z}$ or $C_{z}^{\prime }$ some strictly positive generic constants, $N_{z}$ is a given neighborhood of z, and, for all t∈IR, we define $ES\left(t,z\right)=I\;E\left[Y1_{Y>t}|\;X=z\right]$. Now, to formulate our main results, we will use the hypotheses listed below:

(P1) P(X∈B(z,r))=ϕ(z,r)>0 where $B\left(z,r\right)=\left\{x^{\prime }\in F\;:d\left(z^{\prime },z\right)<r\right\}$.
(P2) $\exists \delta >0,\forall \left(t_{1},t_{2}\right)\in \left[{REXP}_{p}\left(z\right)-\delta ,\;{REXP}_{p}\left(z\right)+\delta \right]$, $\forall \left(z_{1},z_{2}\right)\in N_{x}^{2}$,
  $$|ES\left(t_{1},z_{1}\right)-ES\left(t_{2},z_{2}\right)|\leq C_{x}\left(d^{b}{\left(z_{1},z_{2}\right)}^{b}+\left|t_{1}-t_{2}\right|\right),\;b>0.$$
(P3) The sequence ${\left(X_{i},Y_{i}\right)}_{i\in I\;N}$ is a strong mixing process that has a coefficient α(n) and satisfies $\exists a>2,\exists c>0:\;\forall n\in I\;N,\;\alpha \left(n\right)\leq cn^{-a}$ and
  $$\left\{\begin{array}{c} \forall i\neq j,\;\forall t\in \left[\theta_{x}-\delta ,\theta_{x}+\delta \right],\;I\;E\left[Y_{i}Y_{j}|X_{i},X_{j}\right]\leq C<\infty ,\;\\ I\;P\left(\left(z_{i},X_{j}\right)\in B\left(z,r\right)\times B\left(z,r\right)\right)=\varphi \left(z,r\right)>0.\\ I\;E\left[{\left|Y\right|}^{2}|X\right]<C<\infty \;\mathrm{and}\;I\;E\left[{\left|Y\right|}^{p}\right]<C<\infty ,\;p>1 \end{array}\right.$$
(P4) F is a function with support (0,1) such that
  $$0<C{𝟙}_{\left(0,1\right)}<F\left(t\right)<C^{\prime }{𝟙}_{\left(0,1\right)}<\infty .$$
(P5) There exists a sequence of positive real numbers $\gamma_{n}$ and η>0 such that
  $$\left\{\begin{array}{c} \sum_{n}n^{3p/2}\gamma_{n}^{-p}<\infty ,\\ \sum_{n}n^{\frac{2-a}{a}-\eta }{\left(\chi \left(z,r\right)\right)}^{-(a+4)}<\infty , \end{array}\right.$$
  where $\chi \left(z,r\right)=\max(\varphi \left(z,r\right),\phi^{2}\left(z,r\right))$

*Comments on the hypotheses.*

All these conditions are standards in FTSA. In particular, condition (P1) is checked for several continuous time processes. We refer to [32] for a general Gaussian process viewed as functional space in $L^{2}$. This condition relates the functional structure of the data to the probablity measure of the random variable, measuring the concentration of the probability measure of *X* over a topological ball constructed from the semimetric *d*. At this stage, the function ϕ is affected by two principal factors that are the probability measure and the semimetric *d*. Such assumption can be viewed as generalization for the multivariate case ($X\in R^{k}$) when $\phi \left(z,r\right)=f_{X}\left(z\right)r^{k}+o\left(r^{k}\right)$ where $f_{X}$ is the density of *X*. In this situation, the function ϕ is positive as long as the density $f_{X}$ is structurally positive. A mild regularity condition (P2) is assumed for the distribution function. Such a condition allows for characterizing the nonparametric path of the studied model. (P2) is used to evalaute the bias term of the estimator. Condition (P3) defines the mixing structure of our FTSA framework. The first part of this condition allows for obtaining a convergence rate comprable to the independent case. In conclusion, we can say that the assumed conditions (P1)–(P4) are sufficiently weak to obtain an improved convergence rate, which is comparable to the ideal situation that is the independent case. Of course, these assumptions can be reduced if we are interested in only the convergence estimator without a convergence rate.

Now, we state the following results.

**Theorem** **1.** *Under the suppositions (P1)–(P5), we have*

(2) $$\left|\hat{REX_{p}}\left(z\right)-REX_{p}\left(z\right)\right|=O\left(r^{b}+\sqrt{\frac{\chi^{1/2}\left(z,r\right)\ln n}{n\phi^{2}\left(z,r\right)}}\right).\;a.co.$$

**Proof** **of** **Theorem** **1** For t∈IR, we define

$$\hat{ES}\left(t,z\right)=\frac{\sum_{i=1}^{n}F\left[r^{-1}d\left(z,X_{i}\right)\right]Y_{i}1_{Y_{i}>t}}{\sum_{i=1}^{n}F\left[r^{-1}d\left(z,X_{i}\right)\right]}.$$

So,

$$\hat{ES}\left({\hat{REXP}}_{p}\left(z\right),z\right)=\hat{REX_{p}}\left(z\right),\;and\;ES\left({REXP}_{p}\left(z\right),z\right)=REX_{p}\left(z\right).$$

Then,

$$\hat{REX_{p}}\left(z\right)-REX_{p}\left(z\right)=\hat{ES}\left({\hat{REXP}}_{p}\left(z\right),z\right)-ES\left({\hat{REXP}}_{p}\left(z\right),z\right)$$

$$+ES\left({\hat{REXP}}_{p}\left(z\right),z\right)-ES\left({REXP}_{p}\left(z\right),z\right).$$

Thusly,

$$|\hat{REX_{p}}\left(z\right)-REX_{p}\left(z\right)|\leq \underset{t\in [{REXP}_{p}\left(z\right)-\delta ,\;{REXP}_{p}\left(z\right)+\delta ]}{\sup}\left|\hat{ES}\left(t,z\right)-ES\left(t,z\right)\right|$$

$$\;\;\;+C|{\hat{REXP}}_{p}\left(z\right)-{REXP}_{p}\left(z\right)|.$$

So, Theorem 1 comes from

(3) $$\underset{t\in [{REXP}_{p}\left(z\right)-\delta ,\;{REXP}_{p}\left(z\right)+\delta ]}{\sup}\left|\hat{ES}\left(t,z\right)-ES\left(t,z\right)\right|=O\left(r^{b}+\sqrt{\frac{\chi^{1/2}\left(z,r\right)\ln n}{n\phi^{2}\left(z,r\right)}}\right)\;a.co.$$

and

(4) $$|{\hat{REXP}}_{p}\left(z\right)-{REXP}_{p}\left(z\right)|=O\left(r^{b}+\sqrt{\frac{\chi^{1/2}\left(z,r\right)\ln n}{n\phi^{2}\left(z,r\right)}}\right)\;a.co.$$

The result (4) is proved in [10]. So, we concentrate only on (3). Indeed, as $I\;E\left[{\hat{ES}}_{D}\left(z\right)\right]=1$, we have, for t∈IR,

$$\hat{ES}\left(t,z\right)-\hat{ES}\left(t,z\right)=\frac{1}{{\hat{ES}}_{D}\left(z\right)}[({\hat{ES}}_{N}\left(t,z\right)-I\;E\left[{\hat{ES}}_{N}\left(t,z\right)\right])$$

$$\;\;-(\hat{ES}\left(t,z\right))-I\;E\left[{\hat{ES}}_{N}\left(t,z\right)\right])]-\frac{{\hat{ES}}_{N}\left(t,z\right)}{{\hat{ES}}_{D}\left(z\right)}\left[{\hat{ES}}_{D}\left(z\right)-I\;E\left[{\hat{ES}}_{D}\left(z\right)\right]\right]$$

The proof is carried out through Lemmas 1–3.   □

**Lemma** **1.** *Under the suppositions (P1) and (P3)–(P5), we have*

$${\hat{ES}}_{D}\left(z\right)-I\;E\left[{\hat{ES}}_{D}\left(z\right)\right]=O\left(\sqrt{\frac{\chi^{1/2}\left(z,r\right)\ln n}{n\phi^{2}\left(z,r\right)}}\right)\;a.co.$$

*Moreover,*

$$\sum_{n}I\;P\left({\hat{ES}}_{D}\left(z\right)<\frac{1}{2}\right)<\infty .$$

**Lemma** **2.** *Under the suppositions (P1)–(P2) and (P4)–(P5), we have*

$$\underset{t\in [{REXP}_{p}\left(z\right)-\delta ,\;{REXP}_{p}\left(z\right)+\delta ]}{\sup}\left|ES\left(t,z\right)-I\;E\left[{\hat{ES}}_{N}\left(t,z\right)\right]\right|=O\left(a^{b}\right).$$

**Lemma** **3.** *Under the suppositions (P1)–(P5), we have*

$$\underset{t\in [{REXP}_{p}\left(z\right)-\delta ,\;{REXP}_{p}\left(z\right)+\delta ]}{\sup}\left|{\hat{ES}}_{N}\left(t,z\right)-I\;E\left[{\hat{ES}}_{N}\left(t,z\right)\right]\right|=O\left(\sqrt{\frac{\chi^{1/2}\left(z,r\right)\ln n}{n\phi^{2}\left(z,r\right)}}\right),\;a.co.$$

## 4. Empirical Analysis

This section is devoted to examining the practical use of the model studied in this work. This section is divided into three sections. In the first subsection, we discuss the selection of the smoothing parameter, which is the pivotal parameter in our estimation. For this reason, the choice of this parameter is primordial for the computational aspect. After the smoothing parameter selection, we examine the usefulness of the estimator. This practical study is conducted using two examples. The first one concerns artificial data, and the second one treats financial real data coming from some popular index markets according to the Dow–Jones index.

### 4.1. Smoothing Parameter Selection: Cross-Validation

As mentioned above, the choice of the smoothing parameter *r* is crucial in this nonparametric framework. Now, as our estimation procedure is based on the expectile regression, the appropriate cross-validation (CV) rule is the mean squared error. The latter is usual in nonparametric functional data:

(5) $$r_{CV_{opt}}=\arg\min_{r}\sum_{i=1}^{n}{\left(Y_{i}-{\hat{REXP}}_{0.5}\left(z_{i}\right)\right)}^{2}.$$

This rule is motivated by the fact that the conditional mean IE[Y|X] is associated with ${\hat{REXP}}_{p}$ with p=0.5. The popularity of this approach comes from its easy implementation. However, we can employ a more accurate rule that is a generalization of (5). It is explicitly expressed by

(6) $$r_{opt}=\arg\min_{a}\sum_{i=1}^{n}\rho {\left(Y_{i}-{\hat{REXP}}_{p}\left(z_{i}\right)\right)}^{2},$$

where ρ is the scoring function defining ${REXP}_{p}$. The main advantage of this last rule is its dependence on the threshold *p*. Of course, it is very beneficial in this area of financial risk analysis. Indeed, in risk analysis, we are interested in the tail which corresponds to small or large values of *p*. Observe that the challenging issue in expected shortfall is the absence of the scoring function or backtesting measure. Thus, the use of the optimal smoothing parameter associated with the expectile regression is more adequate for the model $\hat{REX_{p}}$. Moreover, this choice reduces the time cost of the companionability of the ES expectile regression.

### 4.2. Artificial Data

In this empirical analysis, we aim to examine the applicability of the constructed estimator $\hat{REX_{p}}$ as well as its behavior for a finite sample. Clearly, the particularity of this work is the treatment of the dependency case. For this aim, we compare the behavior of the estimator $\hat{REX_{p}}\left(z\right)$ over different levels of dependency. Now, for this purpose, we generate artificial data from the functional autoregressive sampling processes. It is well documented that this kind of process has a strong mixing property and allows controlling the effects of this property on the efficiency of the constructed estimator. We point out that the functional autoregressive process is generated using the R-package version 4.3.1 *freqdom.fda* through the routine code *fts.rar*. We point out that this routine code use of the dynamic functional principal component analysis (see [33]) permits simulating the *p*-order functional autoregressive process using the finite dimensional subspace spanned by given basis functions, such as Fourier basis or spline basis. In this artificial study, we employ this routine code to generate a functional autoregressive process of order p=3. Furthermore, the functional regressors ${\left(X_{i}\right)}_{i}$ are generated as

$$X_{i}=\Psi \left(X_{i-1}\right)+\varepsilon_{i},$$

where Ψ is a kernel operator with kernel ψ(·,·) and $\varepsilon_{i}$ is a white noise random variable. So, under this consideration, the functional covariate is

$$X_{i}\left(t\right)=\sum_{k=1}^{p}\int_{0}^{1}\psi_{k}\left(t,s\right)X_{i-k}\left(s\right)ds+\varepsilon_{i}.$$

**Figure 1:**
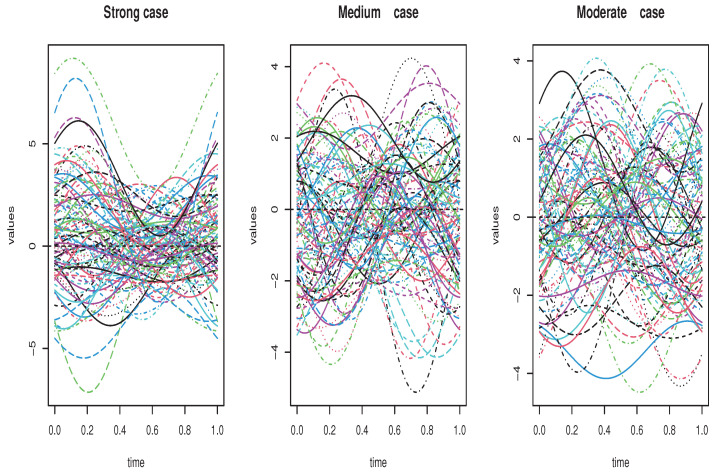
A sample of 100 dependent curves with different lags (colors).

In practice, the kernel operator is associated with the matrix ${\left(\psi_{kij}\right)}_{ij}=\left(<\Psi_{k}\left(v_{i}\right),v_{j}>\right)$. Thus, the dependency level is measured by the values of the coefficients ${\left(\psi_{kij}\right)}_{ij}$. In this empirical analysis, we put $\left(\psi_{kij}\right)=\frac{k+i+j}{k^{2}+i^{2}+j^{2}}$. Moreover, in the routine code *fts.rar*, the operator Ψ should be scaled with respect to its Hilbert–Schmidt norms. The parameter of normalization is so-called *op.norms*. Thus, the dependency degree increases with the value of *op.norms*. In this sense, the great value of *op.norms* implies strong dependency and vice versa. So, in order to examine the effect of the dependency on the estimation quality, we generate three levels of dependencies (strong, medium and moderate correlation). Specifically, the strong dependency is obtained by assuming op.norms = 0.999, while the moderate and the medium cases are, respectively, associated with op.norms = 0.48 and op.norms = 0.01. In Figure 1, we plotted the generated samples of different functional regressors.

Next, the response variable is generated using nonparametric regression formula

$$\begin{array}{ccc} Y_{i} & = & \int_{0}^{1}\sin\left(2+X_{i}^{2}\left(t\right)\right)dt+\int_{0}^{1}\cos\left(2+X_{i}^{2}\left(t\right)\right)dt+\epsilon_{i}, \end{array}$$

where $\epsilon_{i}$ is independent of $X_{i}$, representing the so-called white noise. Obviously, the conditional distribution is related to the distribution of the random variable $\epsilon_{i}$. Thus, to cover different situations, we consider two types of white noise distributions. The first one is normal, which is light-tailed distribution. The second one is Lévy distribution, which is heavy-tailed distribution. We point out that there are several definitions of these notions of light or heavy-tailed distribution. However, the most common index to identify the heavy-tailed distribution is the variance, which should be infinite. Thus, the Lévy distribution is certainly heavy-tailed distribution and has many applications in financial time-series analysis (see [34]). Furthermore, the choice of this distribution is also motivated by the its stability with the linear transformation. If *U* has a standard Lévy distribution Levy(0,1), a+bU is Levy(a,b). For this reason, in both situations (normal or Lévy distribution), the true conditional expected shortfall can be explicitly defined by shifting the distribution of the white noise. In order to highlight the importance of $\hat{REX_{p}}$ in practice, we compare it to the VaR-based expected shortfall, which is estimated by

$$\bar{VEX_{p}}\left(z\right)=\frac{\sum_{i=1}^{n}F\left[r^{-1}d\left(z,X_{i}\right)\right]Y_{i}1_{Y_{i}>{\bar{VaR}}_{p}\left(z\right)}}{\sum_{i=1}^{n}F\left(r^{-1}d\left(z,X_{i}\right)\right)}$$

Of course, the performance of $\bar{VEX_{p}}$ is strongly linked to the estimation of the function $\bar{VaR}$. Thus, in order to provide a more comprehensive comparison with $\hat{REX_{p}}$, we calculate $\bar{VaR}$ with two alternative approaches:

$$\mathrm{The}\;\mathrm{kernel}\;\mathrm{method}\;\hat{VaR}\left(z\right)=\arg\min_{a}\frac{\sum_{i=1}^{n}F\left[r^{-1}d\left(z,X_{i}\right)\right]Q_{p}\left(Y_{i}-a\right)}{\sum_{i=1}^{n}F\left(r^{-1}d\left(z,X_{i}\right)\right)}$$

and

$$\mathrm{the}\;\mathrm{local}\;\mathrm{linear}\;\mathrm{method}\;\tilde{VaR}\left(z\right)=\left(\tilde{a},\tilde{b}\right)*{\left(1,0\right)}^{t}$$

where

$$\left(\tilde{a},\tilde{b}\right)=\arg\min_{a,b}\frac{\sum_{i=1}^{n}F\left[r^{-1}d\left(z,X_{i}\right)\right]Q_{p}\left(Y_{i}-a-bd\left(X_{i},z\right)\right)}{\sum_{i=1}^{n}F\left(r^{-1}d\left(z,X_{i}\right)\right)}$$

and $Q_{p}\left(z\right)=\left(2p-1\right)\left(Y-z\right)+\left|Y-z\right|$. Furthermore, we denote by $\hat{VEX_{p}}$ the estimator associated with the kernel estimator $\hat{VaR}$, while $\tilde{VEX_{p}}$ denotes the estimator associated with the local linear approach.

Clearly, the applicability of all these estimators $\hat{REX_{p}}$, $\hat{VEX_{p}}$ and $\tilde{VEX_{p}}$ rests on the easy selection of different parameters defining these estimators. At this stage, we choose the bandwidth parameters *r*, the semi-metric *d* and the kernel F according to the principal assumptions. For this empirical analysis, we select the optimal bandwidth by the mean square cross-validation rule. The optimization method is performed over a discrete set defined by the $k^{th}$-distance from the location point z where *k* is an integer number that belongs in {5,10,15,20,25,30,…50}. For the kernel F, we choose the β-kernel on (0,1), which is adequate with this kind of nonparametric approach. We point out that this kernel incorporates the technical assumption (P4). On the other hand, the metric choice is closely related to the nature of the functional variable and its smoothing property. It appears that the PCA metric is more suitable for this type of discontinuous functional regressor.

Finally, the performance of both estimators is evaluated by computing

$$Mse=\frac{1}{n}\sum_{i=1}^{n}{\left(Mo\left(X_{i}\right)-\hat{Mo}\left(X_{i}\right)\right)}^{2}{𝟙}_{Y_{i}>{\hat{Mb}}_{p}\left(X_{i}\right)}.$$

where Mo (resp. Mb) means either expectile-based shortfall or VaR-based (respectively, expectile or VaR).

So, for this comparative study, we report estimation error Mse in different situations (degrees of dependency, types of conditional distribution (heavy-tailed and light-tailed)). The results are reported in Table 1.

It is clear that the efficiency of the three estimators is strongly affected by the different axes of this study such as the dependency degree, the nature of the model, as well as the conditional distribution case. In particular, the performance of the three estimators decreases with the level of dependency. On the other hand, it appears clearly that the type of the conditional distribution affects also the behavior of the three estimators. However, it appears that this sensitivity is not very important for the VaR-based expected shortfall $\hat{VEX_{p}}$ and $\tilde{VEX_{p}}$, since the variability of $\hat{VEX_{p}}$ and $\tilde{VEX_{p}}$ is small compared to the $\hat{REX_{p}}$. This conclusion confirms the high sensitivity of the expectile to the extreme values, which is very beneficial for risk management.

### 4.3. Real Data Application

**Figure 2:**
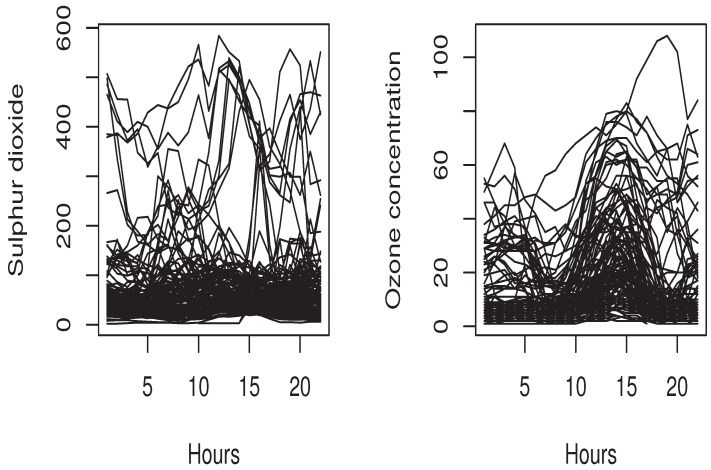
The SO_2_ and O_3_ daily curves.

This section is devoted to the applicability of our model in real time-series data. Specifically, we compare the efficiency of the new ES expectile model to the standard one using environmental time-series data. Although the financial time-series data are the main area where one can use the ES model, the environmental area is also an interesting applied domain of risk management. In fact, the air quality has a important impact on the quality of life. In particular, it is well known that exposures to ground ozone for a period of more than 8 h impact the pulmonary functions as well as the tissues of the respiratory tract. Therefore, it is very important to control the excessive level of ozone concentration. In this context, the theory of the extreme values has been employed to fit this issue. We cite, for instance, [35] and the references therein that use some financial tools to model the risk of air quality. We point out that the standard expected shortfall model is based on the quantiles function, which can be viewed as tail probabilities, while the expectile is tail expectation. The definition of the quantile measures only the frequency of the risk, but the expectile measures the risk frequency and its severity. For this reason, the expectile seems more informative since it has a high sensitivity to the extreme values, which is very beneficial in risk analysis. It permits better detection of the risk of excessive levels of ozone concentration. In this real data analysis, we use the air quality data of the website https://dataverse.harvard.edu/dataverse/beijing-air (accessed on 20 April 2024). It concerns the air quality in Beijing in the northeast of the Chinese country. We focus on two important indices of air quality: sulfur dioxide (SO_2_) and the ozone concentration (O_3_). Recall that (SO_2_) and the ultraviolet rays have a great impact on the stratospheric ozone. So, the functional sample is defined as $X_{i}$, the $i^{th}$-daily curve of SO_2_, and by $Y_{i}$, the total ozone measured on the $i+1^{th}$-day. The sulfur dioxide and the ozone concentration curves are shown in Figure 2.

As discussed below, the principal aim of this real data analysis is to compare the ES expectile regression $\hat{CEX_{p}}$ and the expected shortfall based on the quantile regression, which is defined by

$$V_{p}\left(z\right)=\inf\left\{z\in I\;R:F(z:z)\geq p\right\},$$

where *F* is the conditional cumulative function of *Y* given X=z. We point out that the conditional cumulative distribution function is estimated by

$$\hat{F}\left(z:z\right)=\frac{\sum_{i=1}^{n}1\;I_{(Y_{i}-z)<0}F\left(\frac{d(z,X_{i})}{r_{n}}\right)}{\sum_{i=1}^{n}F\left(\frac{d(z,X_{i})}{r_{n}}\right)}.$$

Then, the Value-at-Risk function is defined

$${\hat{V}}_{p}\left(z\right)=\inf\left\{z\in I\;R:\hat{F}\left(z:z\right)\geq p\right\}.$$

**Figure 3:**
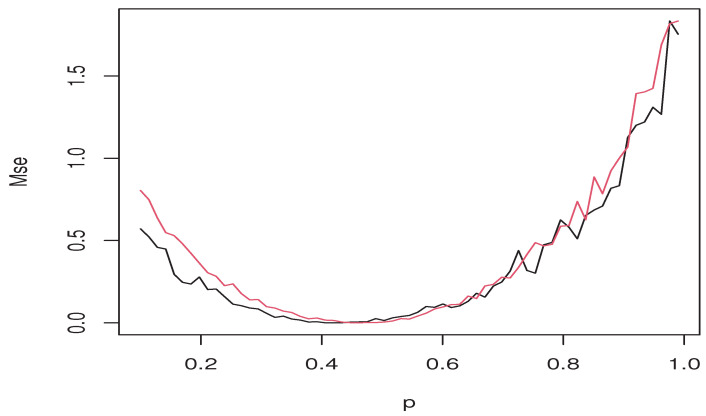
Comparison between ES expectile and ES quantile using Mse.

Thus, the kernel estimator of the standard expected shortfall regression is

$$\tilde{RES_{p}}\left(s\right)=\frac{\sum_{i=1}^{n}F(r^{-1}d\left(z,X_{i}\right)\left(rG\left(r^{-1}\left(\hat{V_{p}}\left(s\right)-Y_{i}\right)\right)+Y_{i}\left(1-H(r^{-1}\left(\hat{V_{p}}\left(s\right)-Y_{i})\right)\right)\right)}{p\sum_{i=1}^{n}K\left(r^{-1}d\left(z,X_{i}\right)\right)},$$

where $G\left(s\right)=\int_{s}^{\infty }uF\left(u\right)$ and $H\left(s\right)=\int_{-infty}^{s}F\left(u\right)du.$ So, our aim is to compare the $\hat{REX_{p}}$ and $\tilde{RES_{p}}$ using real data ${\left(X_{i},Y_{i}\right)}_{i=1,\ldots 365}$. Both estimators are calculated using the same techniques as the empirical analysis section. Specifically, we execute the estimators by the same kernel, select the smoothing parameters by the rule (5), and employ the metric obtained by the PCA metric. We return to Ferraty and Vieu [36] for more details on the mathematical formulation of these metrics. The performance of both estimators is evaluated by computing

$$Mse\left(p\right)=\frac{1}{n}\sum_{i=1}^{n}{\left(Y_{i}-\hat{\Theta_{p}}\left(X_{i}\right)\right)}^{2}{𝟙}_{Y_{i}>\hat{REX_{p}}\left(X_{i}\right)}$$

where $\hat{\Theta_{p}}$ represents $\hat{REX_{p}}$ and $\tilde{RES_{p}}$. Such an error is evaluated as a function of *p*. In Figure 3, we show the values of Mse of both estimators $\hat{REX_{p}}\left(blackline\right)$ and $\tilde{RES_{p}}$ (red line).

The graphs show the superiority of the ES expectile regression over the ES quantile model. In several cases, the black line is under the red line. This superiority is confirmed by reporting the Mse of some *p*. The values of this are given in the following Table 2.

It appears that the ES expectile is more accurate for various values of *p*, giving $\hat{REX_{p}}$ greater precedence as a risk metric.

## 5. Conclusions and Prospects

In this work, we have investigated the free-parameter estimation of the regression of the ES expectile. We have constructed an estimator by the kernel-smoothing approach. This contribution covers the functional time-series case. The theoretical part of this work focuses on the establishment of the convergence in Borell–Contelli in pointwise performance under strong mixing assumptions. This theoretical devolvement constitutes good mathematical support for the use of the new risk metric in risk management. Moreover, the obtained asymptotic results were established under standard conditions and with the precision of the convergence rate. In practice, the applicability of the estimator is very easy and gives better results compared to the standard one. Specifically, we applied the new model for environmental time-series data. The result confirms the superiority of the ES expectile over the ES quantile. This superiority is confirmed in two directions: The first one is the fact that the ES expectile has a small error compared to the ES quantile. The second one is the variability of the error in the ES expectile, which proves its high sensitivity to outliers. This feature is very important in risk analysis, because the risk is often located in the extreme values. Therefore, the robustness of the qunatile is not beneficial in this kind of area. For this reason, the ES expectile is more adequate than the ES quantile. The importance of our contribution can be viewed, also, through the numerous opens questions for the future. For instance, we will treat more dependent cases such as the quasi-associated case or the spatial case. Let us point out that the mixing assumption is very difficult to handle in practice. Thus, this condition can be considered as the principal practical limitation of the present contribution. For this reason, treating other type correlations is very important in practice. It allows for controlling alternative financial time-series data that are not difficult to handle in practice. In addition, the determination of the uniform UNN convergence of the estimator is also a very important prospect in the future. It permits resolving the problem of the smoothing parameter selection. Furthermore, we can also estimate the model using the additive or the linear case.

## 6. The Demonstration of Asymptotic Results

This section is devoted to the proofs of our results. To do that, we start by recalling the principal inequalities used to prove the intermediate lemmas:

**Lemma** **4** ([36])**.** *Let ${\left(Z_{i}\right)}_{i\in N}$ be an α-mixing process. For k∈N, consider two random variables T and $T^{\prime }$ measurable on $\sigma (Z_{i},-\infty <i\leq k)$ and $\sigma (Z_{i},n+k\leq i\leq +\infty )$, respectively:*

*(1)*  *If T and $T^{\prime }$ are bounded, then*
  $$\exists \;C>0,\;cov\left(T,T^{\prime }\right)\leq C\;\alpha \left(n\right).$$
*(2)*  *If there exist three positive integers p, q and r, such that $p^{-1}+q^{-1}+r^{-1}=1$ and $I\;E[T^{p}]<\infty$ and $I\;E[{T^{\prime }}^{q}]<\infty$, then*
  $$\exists \;C>0,\;cov\left(T,T^{\prime }\right)\leq C\;I\;E{\left[T^{p}\right]}^{1/p}\;I\;E{\left[{T^{\prime }}^{q}\right]}^{1/q}\;\alpha {\left(n\right)}^{1/r}.$$

**Lemma** **5** ([36])**.** *Let ${\left(Z_{i}\right)}_{i\in N}$ be an algebraic α-mixing process, which is identically distributed:*

*(1)*  *If there exist p>2 and M>0 such that for all $t>M,\;I\;P(|Z_{1}\left|>t\right)\leq t^{-p}$, then for all r≥1, ϵ>0 and*
  $$I\;P\left(\left|\sum_{i=1}^{n}Z_{i}\right|>\epsilon \right)\leq C\left[{\left(1+\frac{\epsilon^{2}}{rs_{n}^{2}}\right)}^{-r/2}+n\;r^{-1}{\left(\frac{r}{\epsilon }\right)}^{\left(a+1)p/(a+p\right)}\right].$$
*(2)*  *If there exist M<∞ such that $|Z_{1}|\leq M$, then for all r≥1 and C<∞:*
  $$I\;P\left(\left|\sum_{i=1}^{n}Z_{i}\right|>\epsilon \right)\leq C\left[{\left(1+\frac{\epsilon^{2}}{rs_{n}^{2}}\right)}^{-r/2}+n\;r^{-1}{\left(\frac{r}{\epsilon }\right)}^{a+1}\right],$$
  *where $s_{n}^{2}=\sum_{i=1}^{n}\sum_{j=1}^{n}\left|cov\left(Z_{i},Z_{j}\right)\right|$.*

**Proof** **of** **Lemma** **1.** We put

$${\hat{ES}}_{D}\left(z\right)=\frac{1}{n}\sum_{i=1}^{n}\frac{F_{i}}{I\;E\left[F_{1}\right]},$$

We use the Fuck–Nagaev inequality (Lemma 5) to obtain ∀ℓ>0 and ε>0,

(7) $$\begin{array}{ccc} I\;P\left\{\left|I\;E\left[{\hat{ES}}_{D}\left(z\right)\right]-{\hat{ES}}_{D}\left(z\right)\right|>\varepsilon \right\} & = & I\;P\left\{\left|\frac{1}{nI\;E\left[F_{1}\right]}\sum_{i=1}^{n}F_{i}\right|>\varepsilon \right\}\\ & \leq & I\;P\left\{\left|\sum_{i=1}^{n}F_{i}\right|>\varepsilon nI\;E\left[F_{1}\right]\right\}\\ & \leq & C(A_{1}+A_{2}) \end{array}$$

with

$$A_{1}={\left(1+\frac{\varepsilon^{2}n^{2}{\left(I\;E\left[F_{1}\right]\right)}^{2}}{S_{n}^{2}\ell }\right)}^{-\ell /2}\;\mathrm{and}\;A_{2}=n\ell^{-1}{\left(\frac{\ell }{\varepsilon nI\;E[F_{1}]}\right)}^{a+1}$$

where

$$S_{n}^{2}=\sum_{i=1}^{n}\sum_{j=1}^{n}Cov\left(F_{i},F_{j}\right)=S_{n}^{2*}+nVar\left[F_{1}\right]$$

and

$$S_{n}^{2*}=\sum_{i=1}^{n}\sum_{i\neq j}Cov\left(F_{i},F_{j}\right).$$

Now, we must determine the asymptotic term of $S_{n}^{2*}$. For this, we apply the technique of [37] So, we define

$$S_{1}=\left\{\left(i,j\right)\;\;\mathrm{such}\;\mathrm{that}\;\;1\leq i-j\leq m_{n}\right\}$$

and

$$S_{2}=\left\{\left(i,j\right)\;\;\mathrm{such}\;\mathrm{that}\;\;m_{n}+1\leq i-j\leq n-1\right\}$$

with $m_{n}\to \infty ,\mathrm{as}n\to \infty .$ Denote by $J_{1,n}$ and $J_{2,n}$ the covariance sum over $S_{1}$ and $S_{2}$, respectively. Then,

$$J_{1,n}=\sum_{S_{1}}|Cov\left(F_{i},F_{j}\right)|\leq \sum_{S_{1}}\left|I\;E\left[F_{i}F_{j}\right]-I\;E\left[F_{i}\right]I\;E\left[F_{j}\right]\right|.$$

Because of (P1), (P3) and (P5), we can write

$$J_{1,n}\leq Cnm_{n}\chi \left(z,r\right).$$

Now, the covariance over $S_{2}$, can be treated using Davydov–Rio’s inequality (see Lemma 4). Thus, for all i≠j, to

$$|Cov\left(F_{i},F_{j}\right)|\leq C\alpha (|i-j|).$$

Therefore, using $\sum_{j\geq x+1}j^{-a}\leq \int_{u\geq x}u^{-a}={\left[\left(a-1\right)x^{a-1}\right]}^{-1}$ we obtain

(8) $$\left|J_{2,n}\right|=\left|\sum_{\left(i,j\right)\in E_{2}}Cov\left[F_{i},F_{j}\right]\right|\leq C\frac{nm_{n}^{-a+1}}{a-1}.$$

Choosing $m_{n}={\left(\chi (z,r)\right)}^{-1/a}$, we obtain

$$\sum_{i\neq j}^{n}Cov\left(F_{i},F_{j}\right)=O\left(n\chi^{(a-1)/a}\left(z,r\right)\right).$$

Now, the variance part is

$$Var\left(F_{1}\right)\leq C\left(\phi \left(z,r\right)+{\left(\phi \left(z,r\right)\right)}^{2}\right)\leq C\chi^{1/2}\left(z,r\right).$$

Finally, as a>2,

(9) $$S_{n}^{2}=O\left(n\chi^{1/2}\left(z,r\right)\right).$$

Therefore, $\varepsilon =\lambda \frac{\sqrt{S_{n}^{2}\ln n}}{nI\;E[F_{1}]}$ and $\ell =C{\left(\ln n\right)}^{2}$. Thus,

$$A_{2}\leq Cn^{1-(a+1)/2}\chi {\left(z,r\right)}^{-(a+1)/4}{\left(\ln n\right)}^{(3a-1)/2}.$$

Next, from (P5),

$$A_{2}\leq Cn^{-1-\eta (a+1)/2}{\left(\ln n\right)}^{(3a-1)/2}.$$

So, ∃ν>0 such that

(10) $$A_{2}\leq Cn^{-1-\nu }.$$

By (9),

$$A_{1}\leq C{\left(1+\frac{\lambda^{2}\ln n}{\ell }\right)}^{-\ell /2}=C\exp\left(-\ell /2\ln\left(1+\frac{\lambda^{2}\ln n}{\ell }\right)\right)$$

Since $\ell =C{\left(\ln n\right)}^{2}$, we obtain

$$A_{1}\leq C\exp\left(-\lambda^{2}\frac{\ln n}{2}\right)=Cn^{-\lambda^{2}/2}.$$

Thus, for large λ,

(11) $$\exists \nu^{\prime }>0,\;A_{1}\leq Cn^{-\lambda^{2}/2}\leq Cn^{-1-\nu^{\prime }}.$$

In conclusion, for large $C\eta^{2}>1$,

$$\sum_{n}I\;P\left(\left|{\hat{ES}}_{D}\left(z\right)-I\;E\left[{\hat{ES}}_{D}\left(z\right)\right]\right|>\eta \sqrt{\frac{\chi^{1/2}\left(z,r\right)\ln n}{n\phi^{2}\left(z,r\right)}}\right)<\infty .$$

Moreover,

$$\sum_{n\geq 1}I\;P\left(\left|{\hat{ES}}_{D}\left(z\right)\right|\leq 1/2\right)\;\leq \sum_{n\geq 1}I\;P\left(\left|{\hat{ES}}_{D}\left(z\right)-I\;E\left[{\hat{ES}}_{D}\left(z\right)\right]\right|>1/2\right)<\infty .$$

□

**Proof** **of** **Lemma** **2.** Using

$$ES\left(t,x\right)-I\;E\left[{\hat{ES}}_{N}\left(t,z\right)\right]=\frac{1}{I\;E\left[F_{1}\left(z\right)\right]}I\;E\left[F_{1}\left(z\right){𝟙}_{B(z,r)}\left(z_{1}\right)\left(ES\left(t,x\right)-ES\left(t,X_{1}\right)\right)\right].$$

Condition (P2) gives

$${𝟙}_{B(z,r)}\left(z_{1}\right)\left|ES\left(t,x\right)-ES\left(t,X_{1}\right)\right|\leq Cr^{b}.$$

So,

$$\underset{t\in [{REXP}_{p}\left(z\right)-\delta ,\;{REXP}_{p}\left(z\right)+\delta ]}{\sup}\left|ES\left(t,x\right)-I\;E\left[{\hat{ES}}_{N}\left(t,z\right)\right]\right|\leq Cr^{b}.$$

allowing

$$\underset{t\in [{REXP}_{p}\left(z\right)-\delta ,\;{REXP}_{p}\left(z\right)+\delta ]}{\sup}\left|ES\left(t,x\right)-I\;E\left[{\hat{ES}}_{N}\left(t,z\right)\right]\right|=O\left(r^{b}\right)$$

□

**Proof** **of** **Lemma** **3.** Since $\left[{REXP}_{p}\left(z\right)-\delta ,\;{REXP}_{p}\left(z\right)+\delta \right]$ then by the compactness feature we obtain

(12) $$\left[{REXP}_{p}\left(z\right)-\delta ,\;{REXP}_{p}\left(z\right)+\delta \right]\subset \overset{l_{n}}{\underset{j=1}{⋃}}]y_{j}-d_{n},y_{j}+d_{n}[$$

for $d_{n}=O\left(\frac{1}{\sqrt{n}}\right)$ and $l_{n}=O\left(\sqrt{n}\right)$. The two functions $I\;E[{\hat{ES}}_{N}\left(\cdot ,z\right)]$ and ${\hat{ES}}_{N}\left(\cdot ,z\right)$ are increasing. Thus, for $1\leq j\leq l_{n}$,

$$I\;E{\hat{ES}}_{N}(\left(y_{j}-d_{n},z\right)\leq \underset{t\in ]y_{j}-d_{n},y_{j}+d_{n}[}{\sup}I\;E{\hat{ES}}_{N}\left(t,z\right)\leq I\;E{\hat{ES}}_{N}\left(y_{j}+d_{n},z\right)$$

(13) $${\hat{ES}}_{N}\left(t,z\right)y_{j}-d_{n},z)\leq \underset{t\in ]y_{j}-d_{n},y_{j}+d_{n}[}{\sup}{\hat{ES}}_{N}\left(t,z\right)\leq {\hat{ES}}_{N}\left(y_{j}+d_{n},t\right).$$

Now, by (P2) we obtain

$$\;\forall t_{1},t_{2}\in {REXP}_{p}\left(z\right)-\delta ,\;{REXP}_{p}\left(z\right)+\delta .$$

we have

$$\left|I\;E{\hat{ES}}_{N}\left(t_{1},z\right)-I\;E{\hat{ES}}_{N}\left(t_{2},z\right)\right|\leq C\left|t_{1}-t_{2}\right|.$$

Hence,

$$\;\underset{t\in [{REXP}_{p}\left(z\right)-\delta ,\;{REXP}_{p}\left(z\right)+\delta ]}{\sup}\left|{\hat{ES}}_{N}\left(t,z\right)-I\;E{\hat{ES}}_{N}\left(t,z\right)\right|$$

$$\leq \max_{1\leq j\leq l_{n}}\max_{z\in \{y_{j}-d_{n},y_{j}+d_{n}\}}\left|{\hat{ES}}_{N}\left(z,z\right)-I\;E{\hat{ES}}_{N}\left(z,z\right)\right|+Cd_{n}.$$

Clearly

$$d_{n}=n^{-1/2}=o\left(\sqrt{\frac{\chi^{1/2}\left(z,r\right)\ln n}{n\phi^{2}\left(z,r\right)}}\right).$$

Therefore, it suffices to demonstrate that

$$\max_{1\leq j\leq l_{n}}\max_{z\in \{y_{j}-d_{n},y_{j}+d_{n}\}}\left|{\hat{ES}}_{N}\left(z,z\right)-I\;E{\hat{ES}}_{N}\left(z,z\right)\right|=O\left(\sqrt{\frac{\chi^{1/2}\left(z,r\right)\ln n}{n\phi^{2}\left(z,r\right)}}\right),\;a.co.$$

Then, ∀η>0,

$$\begin{array}{ccc} & & I\;P\left(\max_{1\leq j\leq l_{n}}\max_{z\in \{y_{j}-d_{n},y_{j}+d_{n}\}}\left|{\hat{ES}}_{N}\left(z,z\right)-I\;E{\hat{ES}}_{N}\left(z,z\right)\right|>\eta \sqrt{\frac{\chi^{1/2}\left(z,r\right)\ln n}{n\phi^{2}\left(z,r\right)}}\right)\\ & & \leq 2l_{n}\max_{1\leq j\leq l_{n}}\max_{z\in \{y_{j}-d_{n},y_{j}+d_{n}\}}I\;P\left(\left|{\hat{ES}}_{N}\left(z,z\right)-I\;E{\hat{ES}}_{N}\left(z,z\right)\right|>\eta \sqrt{\frac{\chi^{1/2}\left(z,r\right)\ln n}{n\phi^{2}\left(z,r\right)}}\right). \end{array}$$

It remains to assess

$$I\;P\left(\left|{\hat{ES}}_{N}\left(z,z\right)-I\;E{\hat{ES}}_{N}\left(z,z\right)\right|>\eta \sqrt{\frac{\chi^{1/2}\left(z,r\right)\ln n}{n\phi^{2}\left(z,r\right)}}\right).$$

Indeed,

$${\tilde{F}}_{i}\left(z\right)=\frac{1}{I\;E\left[F_{1}\right]\left(z\right)}\left[F_{i}\left(z\right)Y_{i}{𝟙}_{\left\{Y_{i}\leq z\right\}}-I\;E\left[F_{i}\left(z\right)Y_{i}{𝟙}_{\left\{Y_{1}\leq z\right\}}\right]\right].$$

We write

$$\forall \;\varepsilon >0,\;I\;P\left[|{\hat{ES}}_{N}\left(z,z\right)-I\;E{\hat{ES}}_{N}\left(z,z\right)|>\varepsilon \right]=I\;P\left[\frac{1}{n}\left[\left|\sum_{i=1}^{n}{\tilde{F}}_{i}\left(z\right)\right|>\varepsilon \right]\right]$$

(14) $$I\;P\left(\max_{z\in G_{n}}\left|{\hat{ES}}_{N}\left(z,z\right)-I\;E\left[{\hat{ES}}_{N}\left(z,z\right)\right]\right|>\varepsilon \right)\leq \sum_{z\in G_{n}}I\;P\left(\left|{\hat{ES}}_{N}\left(z,z\right)-I\;E\left[{\hat{ES}}_{N}\left(z,z\right)\right]\right|>\varepsilon \right).$$

Because *Y* is not necessarily bounded, we use the truncation method by introducing

$${\hat{ES}}_{N}^{*}\left(z,t\right)=\frac{1}{nI\;E[F\left(h^{-1}d\left(z,X_{1}\right)\right)]}\sum_{i=1}^{n}F\left(r^{-1}d\left(z,X_{i}\right)\right)Y^{*}$$

with $Y^{*}=Y{𝟙}_{\left(Y<\gamma_{n}\right)}$. Thus, the result is a consequence of the intermediate results

(15) $$d_{n}\max_{z\in G_{n}}\left|I\;E\left[{\hat{ES}}_{N}^{*}\left(z,z\right)\right]-I\;E\left[{\hat{ES}}_{N}\left(z,z\right)\right]\right|=O\left(\sqrt{\frac{\chi^{1/2}\left(z,r\right)\ln n}{n\phi^{2}\left(z,r\right)}}\right),$$

(16) $$d_{n}\max_{z\in G_{n}}\left|{\hat{ES}}_{N}^{*}\left(z,z\right)-{\hat{ES}}_{N}\left(z,z\right)\right|=O_{a.co.}\left(\sqrt{\frac{\chi^{1/2}\left(z,r\right)\ln n}{n\phi^{2}\left(z,r\right)}}\right)$$

and

(17) $$d_{n}\max_{z\in G_{n}}\left|{\hat{ES}}_{N}^{*}\left(z,z\right)-I\;E\left[{\hat{ES}}_{N}^{*}\left(z,z\right)\right]\right|=O_{a.co.}\left(\sqrt{\frac{\chi^{1/2}\left(z,r\right)\ln n}{n\phi^{2}\left(z,r\right)}}\right).$$

We start by proving (15): We have, ∀$z\in G_{n}$

$$\left|I\;E\left[{\hat{ES}}_{N}^{*}\left(z,z\right)\right]-I\;E\left[{\hat{ES}}_{N}\left(z,z\right)\right]\right|\leq C\frac{1}{\phi (z,r)}I\;E\left[\left|Y\right|{𝟙}_{Y\geq \gamma_{n}\}}F\left(r^{-1}d\left(z,X\right)\right)\right].$$

By the Holder inequality, for $\alpha^{\prime }$ and β such that $\frac{1}{\alpha^{\prime }}+\frac{1}{\beta }=1$, and $\alpha^{\prime }=\frac{p}{2}$

$$\begin{array}{ccc} \forall z\in G_{n}\\ I\;E\left[\left|Y\right|{𝟙}_{\left\{Y\geq \gamma_{n}\right\}}F\left(r^{-1}d\left(z,X_{1}\right)\right)\right] & \leq & {I\;E}^{1/\alpha }\left[\left|Y^{\alpha }\right|{𝟙}_{\left\{Y\geq \gamma_{n}\right\}}\right]{I\;E}^{1/\beta }\left[F^{\beta }\left(r^{-1}d\left(z,X_{1}\right)\right)\right]\\ & \leq & \gamma_{n}^{-1}{I\;E}^{1/\alpha }\left[\left|Y^{2\alpha }\right|\right]{I\;E}^{1/\beta }\left[F^{\beta }\left(r^{-1}d\left(z,X_{1}\right)\right)\right]\\ & \leq & \gamma_{n}^{-1}{I\;E}^{1/\alpha }\left[\left|Y^{p}\right|\right]{I\;E}^{1/\beta }\left[F^{\beta }\left(r^{-1}d\left(z,X_{1}\right)\right)\right]\\ & \leq & C\gamma_{n}^{-1}\phi^{1/\beta }\left(z,r\right). \end{array}$$

Thus,

$$d_{n}\max_{z\in G_{n}}\left|{\hat{ES}}_{N}^{*}\left(z,z\right)-I\;E\left[{\hat{ES}}_{N}^{*}\left(z,z\right)\right]\right|\leq n^{1/2}\gamma_{n}^{-1}\phi^{(1-\beta )/\beta }.$$

Finally, (15) is consequence of (P5).

Now, for (16), we use Markov’s inequality to show that $\forall z\in G_{n}$, ∀ϵ>0

$$\begin{array}{ccc} I\;P\left(\left|{\hat{ES}}_{N}^{*}\left(z,z\right)-{\hat{ES}}_{N}\left(z,z\right)\right|>\epsilon \right) & \leq & \sum_{i=1}^{n}I\;P\left(Y_{i}>\gamma_{n}\right)\\ & \leq & nI\;P\left(Y>\gamma_{n}\right)\\ & \leq & n\gamma_{n}^{-p}I\;E\left[Y^{p}\right]. \end{array}$$

Choosing $\epsilon =\epsilon_{0}\left(\sqrt{\frac{\chi^{1/2}\left(z,r\right)\ln n}{n\phi^{2}\left(z,r\right)}}\right)$,

$$d_{n}\max_{z\in G_{n}}I\;P\left(|{\hat{ES}}_{N}\left(z,z\right)-{\hat{ES}}_{N}^{*}\left(z,z\right)|>\epsilon_{0}\left(\sqrt{\frac{\chi^{1/2}\left(z,r\right)\ln n}{n\phi^{2}\left(z,r\right)}}\right)\right)\leq n^{3/2-a}<Cn^{-1-\nu }.$$

Now, we prove (17). Define $z\in G_{n}$,

$$\Lambda_{i}=F_{i}Y_{i}^{*}-I\;E\left[F_{1}Y_{i}^{*}\right].$$

Therefore, ∀ϵ>0

$$\begin{array}{ccc} I\;P\left\{\left|{\hat{ES}}_{N}^{*}\left(z,z\right)-I\;E\left[{\hat{ES}}_{N}^{*}\left(z,z\right)\right]\right|>\varepsilon \right\} & = & I\;P\left\{\left|\frac{1}{nI\;E\left[F_{1}\right]}\sum_{i=1}^{n}\Lambda_{i}\right|>\varepsilon \right\}\\ & \leq & I\;P\left\{\left|\sum_{i=1}^{n}\Lambda_{i}\right|>\varepsilon nI\;E\left[F_{1}\right]\right\}. \end{array}$$

We calculate

$$S_{n}^{\prime 2}=\sum_{i=1}^{n}\sum_{j=1}^{n}Cov\left(\Lambda_{i},\Lambda_{j}\right)=\sum_{i=1}^{n}\sum_{i\neq j}Cov\left(\Lambda_{i},\Lambda_{j}\right)+nVar\left[\Lambda_{1}\right].$$

We define

$$S_{1}^{\prime }=\left\{\left(i,j\right)\;\;\mathrm{such}\;\mathrm{that}\;\;1\leq i-j\leq u_{n}\right\}$$

and

$$S_{2}^{\prime }=\left\{\left(i,j\right)\;\;\mathrm{such}\;\mathrm{that}\;\;u_{n}+1\leq i-j\leq n-1\right\}.$$

Let $J_{1,n}^{\prime }$ and $J_{2,n}^{\prime }$ be the sum of covariance over these two sets, respectively. On $S_{1}^{\prime }$, we have

$$\begin{array}{ccc} J_{1,n}^{\prime } & = & \sum_{S_{1}^{\prime }}\left|Cov(\Lambda_{i},\Lambda_{j})\right|\\ & \leq & C\sum_{S_{1}^{\prime }}\left|I\;E\left[F_{i}F_{j}\right]\right|+\left|I\;E\left[F_{i}\right]I\;E\left[F_{j}\right]\right|. \end{array}$$

Because of (P1), (P3) and (P5), we have

$$J_{1,n}^{\prime }\leq Cnu_{n}\chi \left(z,r\right).$$

By Davydov-Rio’s inequality (see, Lemma 4) in the $L^{\infty }$ cases we have

$$|Cov\left(\Lambda_{i},\Lambda_{j}\right)|\leq C\gamma_{n}^{2}\alpha (|i-j|).$$

Hence,

$$J_{2,n}^{\prime }=\sum_{S_{2}^{\prime }}\left|Cov\left(\Lambda_{i},\Lambda_{j}\right)\right|\leq \frac{n\gamma_{n}^{2}u_{n}^{-a+1}}{a-1}.$$

Choosing $u_{n}={\left(\frac{\gamma_{n}^{2}}{\chi (z,r)}\right)}^{1/a}$, we prove that

$$\sum_{i=1}^{n}\sum_{i\neq j}Cov\left(\Lambda_{i},\Lambda_{j}\right)=O\left(n\gamma_{n}^{2/a}\chi {\left(z,r\right)}^{(a-1)/a}\right).$$

On the other hand,

$$\begin{array}{c} Var\left(\Lambda_{1}\right)\leq I\;E{\left[F_{i}Y_{i}^{*}\right]}^{2}\leq I\;E{\left[F_{i}Y_{i}\right]}^{2}=O\left(\phi \left(z,r\right)\right). \end{array}$$

Thus,

(18) $$S_{n}^{\prime 2}=O\left(n\chi^{\left(1/2\right)}\left(z,r\right)\right).$$

Fuck–Nagaev’s inequality (see Lemma 5) over $\Lambda_{i}$ implies that ∀ℓ>0 and ε>0,

$$\begin{array}{ccc} I\;P\left\{\left|I\;E\left[{\hat{ES}}_{N}^{*}\left(z,z\right)\right]-{\hat{ES}}_{N}^{*}\left(z,z\right)\right|>\varepsilon \right\} & \leq & I\;P\left\{\left|\sum_{i=1}^{n}\Lambda_{i}\right|>\varepsilon nI\;E\left[F_{1}\right]\right\}\\ & \leq & C(A_{1}^{\prime }\left(z\right)+A_{2}^{\prime }\left(z\right)) \end{array}$$

where

$$A_{1}^{\prime }={\left(1+\frac{\varepsilon^{2}n^{2}{\left(I\;E\left[F_{1}\right]\right)}^{2}}{S_{n}^{\prime 2}\ell }\right)}^{-\ell /2}\;\mathrm{and}\;A_{2}^{\prime }=n\ell^{-1}{\left(\frac{\ell }{\varepsilon nI\;E[F_{1}]}\right)}^{a+1}.$$

Taking $\varepsilon =\lambda^{\prime }\frac{\sqrt{n\ln n\chi^{1/2}\left(z,r\right)}}{nI\;E[F_{1}]}$ and $\ell =C{\left(\ln n\right)}^{2}$, we obtain

(19) $$\begin{array}{c} d_{n}A_{2}^{\prime }\leq Cn^{3/2-(a+1)/2}\chi {\left(z,r\right)}^{-(a+1)/4}{\left(\ln n\right)}^{(3a-1)/2}\leq Cn^{-1-\nu_{1}^{\prime }} \end{array}$$

for some $\nu_{1}^{\prime }>0$. Similarly to (11), we prove for $\ell =O{\left(\ln n\right)}^{2}$ that

(20) $$\begin{array}{c} d_{n}A_{1}^{\prime }\leq C{\left(1+\frac{\lambda^{\prime 2}\ln n}{\ell }\right)}^{-\ell /2}\leq Cn^{-1-\nu_{2}^{\prime }}\;\nu_{2}^{\prime }>0. \end{array}$$

Then, (19) and (20) permit concluding. Therefore, for $\tau >\frac{1}{\sqrt{C}}$,

(21) $$\left|{\hat{ES}}_{N}\left(z,z\right)-I\;E{\hat{ES}}_{N}\left(z,z\right)\right|=O_{a.co.}\left(\sqrt{\frac{\chi^{1/2}\left(z,r\right)\ln n}{n\phi^{2}\left(z,r\right)}}\right).$$

□

## Figures and Tables

**Table 1:** MSE error for the estimator for different dependency levels.

Conditional Distribution	Level of Dependency	*p*	$\hat{REX_{p}}$	$\hat{VEX_{p}}$	$\tilde{VEX_{p}}$
Normal distribution	Strong dependency				
		0.01	0.138	0.554	0.446
		0.05	0.125	0.447	0.436
		0.90	0.102	0.428	0.414
		0.95	0.162	0.374	0.367
Normal distribution	Medium dependency				
		0.01	0.098	0.311	0.308
		0.05	0.081	0.302	0.293
		0.90	0.075	0.282	0.176
		0.95	0.093	0.203	0.199
Normal distribution	Moderate dependency				
		0.01	0.049	0.161	0.154
		0.05	0.062	0.181	0.171
		0.90	0.051	0.168	0.160
		0.95	0.073	0.192	0.182
Lévy distribution	Strong dependency				
		0.01	0.610	0.581	0.472
		0.05	0.630	0.532	0.423
		0.90	0.310	0.442	0.309
		0.95	0.280	0.364	0.251
Lévy distribution	Medium dependency				
		0.01	0.290	0.271	0.235
		0.05	0.090	0.182	0.111
		0.90	0.051	0.113	0.102
		0.95	0.154	0.117	0.106
Lévy distribution	Moderate dependency				
		0.01	0.151	0.241	0.192
		0.05	0.128	0.214	0.189
		0.90	0.033	0.217	0.195
		0.95	0.038	0.143	0.117

**Table 2:** Comparison between Mse of ES expectile and ES quantile.

Cases	*p* = 0.99	*p* = 0.5	*p* = 0.1	*p* = 0.05	*p* = 0.01
ES expectile	1.76	0.14	0.53	0.48	0.56
ES quantile	1.79	0.18	0.38	0.68	0.88

## Data Availability

The data used in this study are available through the link https://dataverse.harvard.edu/dataverse/beijing-air (accessed on 20 April 2024).
